# Effects of Zeolite on Virulence Gene Content in Avian Pathogenic *Escherichia coli*

**DOI:** 10.1155/vmi/1983487

**Published:** 2026-06-24

**Authors:** Abdalmajeed M. Alajlouni, Wael Hananeh

**Affiliations:** ^1^ Department of Veterinary Pathology and Public Health, Faculty of Veterinary Medicine, Jordan University of Science and Technology, P.O. Box 3030, Irbid, 22110, Jordan, just.edu.jo

**Keywords:** pathogenic *E*. *coli*, poultry, virulence, zeolite

## Abstract

Avian pathogenic *Escherichia coli* (APEC) continues to be a major concern for public health and animal farming due to its diverse virulence factors that support iron uptake, adherence, toxin release, and resistance to host defenses. While natural zeolites are commonly incorporated into animal feed for their detoxifying, buffering, and adsorption capabilities, little is known about how they affect the presence of pathogenic genes in gut bacteria. This study explored whether feeding broiler chickens with natural zeolite could affect the virulence gene profiles of *E. coli* found in their ceca. One hundred fifty chicks were divided into five groups receiving 0%, 5%, 10%, 15%, or 30% zeolite. Eleven genes were screened using PCR. Results showed that zeolite levels significantly affected the virulence‐associated gene carriage of E. *coli*, with the 10% zeolite group showing no isolates that met the APEC classification criteria (≥ 5 virulence genes) and the lowest presence of minimal predictor virulence genes (*iroN, ompT, iutA, hlyF,* and *iss*), indicating that 10% zeolite was the most effective level under the conditions of this study. These findings mark the first report that natural zeolite can reduce virulence gene carriage in *E. coli* within a live animal model, supporting its potential as a nonantibiotic method to reduce APEC risk and for modulating pathogenic bacterial populations. Further investigation, including gene expression and challenge trials, is needed to better understand the underlying mechanisms and validate its protective benefits.

## 1. Introduction

*Escherichia coli* (*E. coli*) are considered normal inhabitants of the gastrointestinal tract of humans and animals, and they constitute an essential component of the gut microbiome. However, certain strains possess pathogenic potential that allows them to cause a wide range of diseases. The pathogenic ability of *E. coli* strains is facilitated by a broad range of virulence factors, which are coded by virulence‐associated genes (*iutA*, *iss*, *papC*, *iucD*, *tsh*, *irp-2*, *ompT*, *hlyF*, *iroN*, *cva/cvi*, and *astA*). These genes contribute to bacterial adhesion, iron acquisition, serum resistance, toxin production, and immune evasion. According to the genetic criteria, the pathogenicity of avian pathogenic *E*. *coli* (APEC) strains is determined by the presence of at least five virulence genes [1, 2].

Zeolites are naturally occurring volcanic minerals mined in certain parts of the world. They are a group of hydrated aluminosilicates of alkaline and alkaline earth metals, primarily sodium, potassium, magnesium, and calcium, characterized by low mining cost, abundance, low bulk density, and high resistance to alteration [3]. The typical chemical composition of zeolites includes Na_2_O, K_2_O, SiO_2_, Al_2_O_3_, and Fe_2_O_3_, though their relative proportions vary depending on geological origin [4]. The functional properties of different types of zeolites, such as high water‐holding capacity, high cation‐exchange capacity, and high adsorption capacity, depend largely on these compositional differences [5].

Due to their unique physicochemical characteristics, zeolites are widely used across multiple industrial and agricultural sectors. In animal production, they are applied in intensive aquaculture systems for ammonia removal and serve as feed additives in poultry, sheep, and cattle farming [6, 7]. For instance, El‐Nile et al. [8] investigated the effects of zeolite supplementation in natural and nanoform on ruminal fermentation characteristics, milk chemical composition, and milk production of dairy goats. The results revealed that the nanoform of zeolite had more effects as a feed additive than the natural forms [8]. Similarly, supplementation with natural zeolite in laying hens improved eggshell ratio and density [9]. In broiler studies, dietary inclusion of zeolite at concentrations of 0–20 g/kg enhanced production performance up to 10 g/kg, although the effect was not statistically significant [10]. In addition to that, a study performed in Jordan using bentonite nanoparticles to study the effect of zeolite on broiler performance and carcass characteristics revealed that concentrations of 1%, 2%, and 3% can enhance the growth performance of broilers with no change on carcass characteristics [11]. Natural zeolites effectively detoxify economical pollutants, carcinogenic contaminants, and heavy metals and encourage animal welfare and performance at higher growth rates [12]. Zeolites act as microecological modulators. They do not sterilize the gut; rather, they modulate its chemical environment. Their buffering effect helps stabilize pH, creating conditions less favorable to acid‐sensitive pathogens, and over time, these effects may increase beneficial bacteria such as *Lactobacillus* and *Bifidobacterium* while decreasing harmful or opportunistic microbes. A natural zeolite was incorporated into the diets of laying hens at inclusion levels of 1%, 2%, and 4% (w/w) over a 23‐week feeding period. Zeolite supplementation led to a significant reduction in bacteria belonging to Enterobacteriaceae across all tested concentrations, and the result was that dietary zeolite reduced the carriage of several poultry‐associated pathogens while maintaining populations of beneficial gut bacteria [13]. Based on the report of the European Food Safety Authority (EFSA) [14], 2025, natural zeolite is authorized to be used as a binder and anticaking agent in feed for all animal species with a maximum level of 10,000 mg/kg of feed [14]. Although several studies have demonstrated the beneficial effects of zeolite supplementation on animal health and microbial balance, no previous research has examined its impact on the distribution and abundance of pathogenic genes within the gut microbiome. Therefore, the present study was conducted to evaluate the effects of Jordanian natural zeolite supplementation on the pathogenic gene content of *E*. *coli* strains isolated from the intestines of broiler chickens.

## 2. Materials and Methods

### 2.1. Birds and Housing

One hundred fifty broiler chicks (1‐day‐old; mixed sex; 40 ± 2 g) were randomly allocated to 15 floor pens. Temperature was reduced from 32°C ± 1°C to 22°C by Day 21 (RH 55%–65%); lighting was 23L:1D (Week 1) then 20L:4D. Feed and water were provided ad libitum. The study was read and approved by the Institutional Animal Care and Use Committee (IACUC) at JUST under approval number 16/4/12/738.

### 2.2. Experimental Design and Diets

A completely randomized design was employed, consisting of five dietary treatments containing 0%, 5%, 10%, 15%, and 30% zeolite. The zeolite used in this study was a natural form sourced from the Al‐Azraq region of Jordan. The zeolite was milled (< 250 μm) and stored airtight. Phillipsite predominated in all samples. ICP‐MS showed Pb, Cd, As, and Hg below feed additive limits. Moisture (105°C, 4 h) and microbiological counts met feed hygiene standards.

Zeolite was incorporated during diet preparation by replacing an equivalent proportion of the diet according to the respective inclusion levels of each treatment. Each treatment was replicated three times. The supplementation of a wide range of concentrations (0%–30%) was intended to explore a dose–response relationship, including both practical and higher inclusion levels, in order to better understand the potential effects and safety limits of zeolite supplementation.

The experimental diets were based on a corn–soybean meal formulation, formulated to be isonitrogenous and isocaloric, and were designed to meet the nutritional requirements for broiler chickens according to local feeding standards (Table 1). However, the inclusion of high levels of zeolite may have altered the isonitrogenous and isocaloric balance of the diets. Zeolite was incorporated intentionally at these levels to evaluate the potential effects of such substitution under practical feeding conditions.

**TABLE 1:** Poultry feed formulation (percentage composition).

Item	Starter	Grower
Corn	48	52
Soybean meal	37	31
Wheat	10	12.5
Oil	1	1.3
Calcium carbonate	1.3	1.2
Lysine	0.6	0.55
Methionine	0.37	0.35
Threonine	0.16	0.14
Vitamin premix 0.1%	0.1	0.1
Mineral premix 0.1%	0.1	0.1
Choline	0.075	0.075
Sodium bicarbonate	0.15	0.14

*Note:* All values represent the inclusion rate as a percentage of the total diet on an as‐fed basis.

### 2.3. Sample Collection

At the end of the experiment (Day 35), three birds were randomly selected from each pen, ensuring their body weights were closest to the pen’s mean body weight. The selected birds were humanely slaughtered, and their ceca were aseptically collected, transported to the laboratory, and stored at 4°C until further analysis. The cecum was selected because it is the ideal habitat for a diverse microbiome and is indeed the important organ studied for the intestinal microbiome of poultry [15].

### 2.4. Detection of Virulence Genes

Different strains of *E. coli* were isolated from 45 cecum samples of broiler chickens. A total of 102 isolates were obtained (2‐3 per sample) from each cecal sample based on distinct colony morphology (size, shape, and color) on selective media, followed by confirmation using standard biochemical identification methods. Based on the genetic criteria for virulence genes, two multiplex‐PCR reactions (M1 and M2) were done to assess the profile of 11 virulence‐associated genes (Table 2) in the isolates; each reaction contained 4 μL of 5x Firepol Blend Master Mix (Solis BioDyne, Estonia), 1.2 μL of each primer pair at 10 pmol, and up to 20 μL nuclease‐free water. Reaction conditions for the M1 PCR were initial denaturation at 94°C for 10 min followed by 35 cycles of denaturation at 94°C for 30 s, annealing at 63°C for 30 s, extension at 72°C for 30 s, and final extension at 72°C for 10 min, whereas the conditions for the M2 reaction were initial denaturation at 94°C for 10 min followed by 35 cycles of denaturation at 94°C for 30 s, annealing at 58°C for 30 s, extension at 72°C for 60 s, and final extension at 72°C for 10 min. Isolates containing at least five virulence genes were considered the APEC strains, and isolates containing less than five virulence genes were considered the avian nonpathogenic *Escherichia coli* (non‐APEC) strains [18]. PCR procedures were performed under standardized conditions, using positive and negative controls and optimized cycling parameters to ensure specificity and reproducibility of PCR product amplification.

**TABLE 2:** Primers used for multiplex PCR detection of virulence‐associated genes in *E. coli* isolates.

Gene	Primer sequence	Product size	Reaction/annealing	Reference
*ompT*	F: TCATCCCGGAAGCCTCCCTCACTACTAT	496	M1/63°C	[16]
	R: TAGCGTTTGCTGCACTGGCTTCTGATAC			
*hlyF*	F: GGCCACAGTCGTTTAGGGTGCTTACC	450		
	R: GGCGGTTTAGGCATTCCGATACTCAG			
*iroN*	F: AATCCGGCAAAGAGACGAACCGCCT	553		
	R: GTTCGGGCAACCCCTGCTTTGACTTT			
*iutA*	F: GGCTGGACATCATGGGAACTGG	302		
	R: CGTCGGGAACGGGTAGAATCG			

*iss*	F: CAGCAACCCGAACCACTTGATG	323	M2/58°C	[17]
	R: AGCATTGCCAGAGCGGCAGAA			
*papC*	F: TGATATCACGCAGTCAGTAGC	501		
	R: CCGGCCATATTCACATAA			
*iucD*	F: ACAAAAAGTTCTATCGCTTCC	714		
	R: CCTGATCCAGATGATGCTC			
*tsh*	F: ACTATTCTCTGCAGGAAGTC	824		
	R: CTTCCGATGTTCTGAACGT			
*irp-2*	F: AAGGATTCGCTGTTACCGGAC	413		
	R: AACTCCTGATACAGGTGGC			
*cva/cvi*	F: TGGTAGAATGTGCCAGAGCAAG	1181		
	R: GAGCTGTTTGTAGCGAAGCC			
*astA*	F: TGCCATCAACACAGTATATCC	116		
	R: TCAGGTCGCGAGTGACGGC			

*Note:* F, forward primer; R, reverse primer; M1, multiplex PCR reaction 1 (annealing at 63°C); M2, multiplex PCR reaction 2 (annealing at 58°C). Product sizes are given in base pairs (bp).

### 2.5. Statistical Analysis

**FIGURE 1:**
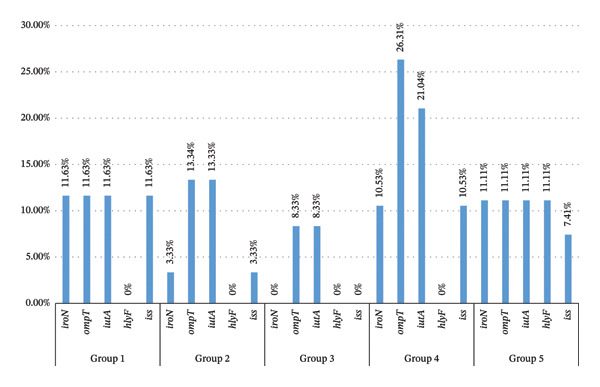
Detection frequency of the five minimal predictor virulence genes (*iroN*, *ompT*, *iutA*, *hlyF*, and *iss*) across treatment groups. Values represent the percentage of isolates within each group that tested positive for each gene. Group 1: 0% zeolite (*n* = 10 isolates); Group 2: 5% zeolite (*n* = 10); Group 3: 10% zeolite (*n* = 10); Group 4: 15% zeolite (*n* = 7); and Group 5: 30% zeolite (*n* = 10).

The experiment was conducted using a completely randomized design, in which dietary treatments were applied at the pen level. A total of 150 broiler chickens were allocated to 15 pens, with three replicate pens per treatment (10 birds per pen). For virulence gene analysis, isolates from three birds per pen were pooled to obtain pen‐level proportions as isolates within a pen are not independent (*n* = 102). The proportion of APEC isolates per pen was analyzed by one‐way ANOVA and Tukey’s post hoc test (SPSS v26.0) with significance set at *p* < 0.05. Figure 1 illustrates the descriptive detection frequencies of individual genes at the isolate level; however, no statistical comparison was made for individual gene frequencies.

## 3. Results

### 3.1. Detection of APEC and Virulence‐Associated Genes

Table 3 reports the number and percentage of isolates classified as APEC (≥ 5 virulence genes) vs. non‐APEC (< 5 genes) per treatment group. Group sample sizes were as follows (number of isolates): Group 1: *n* = 10; Group 2: *n* = 10; Group 3: *n* = 10; Group 4: *n* = 7; and Group 5: *n* = 10. The highest number of non‐APEC was found in Group 3 (100%), followed by Group 2 and Group 5 (80% each), Group 4 (71.4%), and Group 1 (50%)

**TABLE 3:** Distribution of *E. coli* isolates classified as non‐APEC (carrying < 5 virulence genes) and APEC (carrying ≥ 5 virulence genes) across experimental groups.

Group	*E. coli*	
	Non‐APEC	APEC
Group 1	5 (50%)	5 (50%)
Group 2	8 (80%)	2 (20%)
Group 3	10 (100%)	0 (0%)
Group 4	5 (71.4%)	2 (28.6%)
Group 5	8 (80%)	2 (20%)

*Note:* Values represent the number of isolates (percentage of group total). Group sample sizes (total isolates screened per group): Group 1 (0% zeolite), *n* = 10; Group 2 (5% zeolite), *n* = 10; Group 3 (10% zeolite), *n* = 10; Group 4 (15% zeolite), *n* = 7; and Group 5 (30% zeolite), *n* = 10.

Abbreviation: APEC, avian pathogenic *Escherichia coli*.

Table 3 shows that Group 3 consisted entirely of non‐APEC isolates (100%), meaning none of the strains in that group carried the threshold number of virulence genes required for APEC classification. Groups 2 and 5 each contained a high proportion of non‐APEC isolates (80%), suggesting limited virulence potential within those populations. Group 4 displayed a somewhat lower percentage of non‐APEC isolates (71.4%), indicating a mix of strains with varying virulence profiles. In contrast, Group 1 showed the lowest proportion of non‐APEC isolates (50%), implying that half of the strains in this group carried enough virulence genes to be considered potentially pathogenic.

According to the number of minimal predictor virulence genes [16], the lowest number of detected genes occurred in Group 3 (2 out of 5), followed by Groups 4, 2, and 1 (4 out of 5), and lastly, Group 5 (5 out of 5) (Figure 1).

The percentage of the minimal predictor virulence genes (*iroN*, *ompT*, *iutA*, *hlyF*, and *iss*) detected differed among the five groups that were investigated. Group 1 showed the presence of the four virulence genes *iroN*, *ompT*, *iutA*, and *hlyF* at the same frequency (11.67) and no *iss*. A similar trend was observed in Group 2, where the four genes were likely with almost the same frequency (13.33), and no *iss* was detected. The general virulence gene detection was less in Group 3. Only *iroN* (8.33) and *ompT* (3.33) were found. On the other hand, Group 4 had the most prevalent virulence genes of all the groups. It is noteworthy that *iutA* and *hlyF* both had significantly high detection rates of 25.31 and 21.04, respectively, whereas *iroN* and *ompT* had 10.57 and 10.57, respectively. This group did not show the *iss* gene. Group 5 had moderate and comparatively homogeneous detection of the predictor gene with *iroN*, *ompT*, *iutA*, and *hlyF* detected in 11.11% of the isolates.

## 4. Discussion

The present study demonstrates that dietary supplementation with Jordanian natural zeolite significantly modulates the virulence‐associated gene carriage of *E. coli* strains isolated from the ceca of broiler chickens. The most striking finding was the complete absence of isolates meeting the APEC classification criteria (≥ 5 virulence genes) in the broilers fed a 10% zeolite diet (Group 3), with 100% of isolates classified as nonpathogenic. This decrease in virulence gene carriage is consistent with the observed reduction in the number of minimal predictor virulence genes, with isolates from the 10% zeolite group carrying only two of the five key predictor genes. These findings provide the first evidence that zeolite may reduce the genetic determinants associated with APEC in vivo. The mechanisms of this effect are, probably, complex in nature, based on the physicochemical properties of zeolites. However, the present study was not designed to elucidate mechanisms; the following explanations are hypothetical and require direct testing. One of the most influential processes might be the sequestration of iron, which is an important micronutrient to the bacteria and their growth [19]. To overcome the iron‐restricted environment of the host and ensure the availability of this essential metal, APEC strains employ iron acquisition systems encoded by the *iutA*, *iucD*, *irp-2*, and *iroN genes* [20]. Zeolite has the potential to cause a condition of iron restriction in the gut microenvironment by binding iron and lowering its bioavailability [21]. This nutritional stress may be selective to those bacteria that downregulate these energetically costly virulence systems or be selective to allow bacteria that do not possess such systems to grow [22]. This hypothetical correlation of nutritional disarmament from previous studies finds strong support in the lowest evidence of these iron‐related genes found in the 10% zeolite group.

The nonlinear effect that can be observed is most when the zeolite is at 10% but shows a partial offset at 15% and 30%. The possible causes of this reversal are gut overload, which may decrease the level of nutrient digestibility and change gut motility, or excess binding of cations, which might cause an imbalance of other crucial minerals [23]. Based on that, it can be hypothesized that, at very high concentrations, the adsorption and binding capacity of zeolite may exert a selective pressure on the bacterial population, favoring APEC subpopulations with more efficient iron acquisition systems or other adaptive genetic traits. Such adaptations could enhance bacterial survival and persistence despite the nutrient‐restricted environment created by zeolite supplementation. In addition to that, reducing dietary protein decreased the performance and immune capacity of broilers, and that may have positive effects on the APEC [24]. It is worth mentioning that levels of inclusion above 10% changed the diet composition significantly, which may change digestibility of nutrients and gut physiology as a whole. Thus, while 10% had the most influence on virulence gene carriage, application in practice would need careful reformulation to maintain isonitrogenous and isocaloric balance.

Also, the effect of zeolite is not limited to mere nutrient sequestration but is more broadly related to microecology. It is known that pH‐buffering capacity can stabilize the microenvironment of the gut and create less favorable living conditions for acid‐sensitive pathogens like certain *E. coli* strains. In a recent review, it was concluded that numerous studies had illustrated the beneficial impact of zeolite (clinoptilolite) and its micronized and mechanically activated forms on gut health and microbiota due to their detoxifying, anti‐inflammatory, immunostimulating, and antioxidative properties [21]. In chickens, it was reported that dietary inclusion of 1%–4% of zeolite modulated the gut bacterial counts, by which the Enterobacteriaceae counts decreased in laying hens with simultaneously increased counts of other bacteria like *Lactobacillus* and *Bifidobacterium*. It was found that a diet supplemented with 2% zeolite has a positive impact on the health status of *E. coli* O157:H7–infected broilers and enhanced hematological profiles, with efficacy comparable to antibiotic treatment [25].

The current study could have an indirect effect on the virulence gene expression through a certain shift of the gut microbiome, which could happen in two ways. To begin with, a healthier and more competitive microbial community will practice competitive exclusion to restrict the niche and the resources that the pathogens have. Second, this shift can affect the bacterial quorum sensing networks, which are used to regulate virulence gene expression in respect to population density [26]. The decrease in gut lining inflammatory signals would also eliminate triggers that are known to elevate the expression of APEC virulence factors like *ompT*, *hlyF,* and *iss*. Without direct measurements of microbiome composition, quorum sensing molecules, or inflammatory markers, these mechanisms remain speculative.

The zeolite technical results can be generalized to virulence genes, which show that a 10% Jordanian zeolite inclusion rate might be a potentially effective level in promoting growth and reduction of APEC under the specific conditions of this study. It was expected that 30% Jordanian zeolite inclusion would recover all five minimal predictor genes, which reinforced the idea that excess is not necessarily the best and also emphasized the need to optimize the dose. A similar trend was previously reported for a dose‐dependent effect of Jordanian zeolite on broiler weight gain [27]. The 50% APEC prevalence of the control condition (0% zeolite) reflects the reports in the field concerning intensive broiler systems in the developing regions, where colibacillosis has been a primary cause of economic loss [18]. The finding that none of the isolates from the 10% zeolite group fulfilled the criteria of APEC classification indicates a possible nonantibiotic strategy that needs further evaluation under commercial conditions. On a molecular level, zeolite acts as an immunomodulator through substantial upregulation of expression of the wide range of cytokine genes in the intestinal mucosa that includes important interleukins (IL1B, IL4, IL6, IL10, and IL12) and interferons (IFNG and IFNB) [28]. At the same time, it will be possible to promote the integrity of the gut barrier, improving tight junctions of the intestinal mucosa. This twofold action, involving both immune response priming and reinforcement of the physical intestinal barrier, forms the molecular basis for its health effects [28].

Inflammatory and physiological parameters were not directly assessed within the present study; however, parallel toxicological study conducted by Hananeh et al. using the same zeolite source and inclusion levels demonstrated that zeolite supplementation did not induce any significant physiological, hematological, biochemical, or histopathological changes [29], supporting the safety of the tested levels in this model.

In the current research, *E. coli* isolates were classified into APEC and non‐APEC groups based on established virulence gene criteria. Even though this approach provides a practically accepted framework for assessing pathogenic potential, additional classification of non‐APEC strains into additional pathotypes may offer deeper insights into the functional diversity of *E. coli* populations within the poultry gut. Such expanded pathotyping approaches may provide a more comprehensive understanding of potential shifts in bacterial populations in response to dietary interventions. Therefore, their inclusion should be considered in future studies to better elucidate the effects of these interventions on APEC virulence profiles and population dynamics.

The limitation of the current research is that the study evaluated the virulence genes of *E. coli* isolates rather than their expression levels. Future studies including transcriptomic approaches would provide valuable insights into the regulation and expression dynamics of these virulence determinants. Moreover, the study did not measure the actual impact of zeolite supplementation on disease outcomes through challenge experiments or infection models as the reduction in the number of detected virulence‐associated genes may indicate a lower probability of pathogenic potential, as supported by previous studies, but does not exclude pathogenicity. Finally, the classification of isolates was limited to APEC and non‐APEC categories, so further characterization into additional *E. coli* pathotypes should be considered within future investigations. Importantly, virulence‐associated genes were evaluated in this study and not actual virulence, pathogenicity, or disease outcome. The decrease in gene detection indicates a lower genetic potential for virulence, but challenge studies are required to confirm whether this will result in reduced disease susceptibility.

In conclusion, the dietary inclusion of Jordanian natural zeolite at 10% was associated with a significant reduction in virulence‐associated gene carriage, and no isolates from this group fulfilled the APEC classification criteria. It is hypothesized that this effect may involve iron sequestration and pH alterations; however, direct mechanistic evidence is lacking. These findings warrant further investigation into gene expression profiling (qPCR) and challenge studies to confirm reduced disease susceptibility. If validated, zeolite supplementation could emerge as a sustainable, nonantibiotic intervention to mitigate APEC‐related losses in poultry production and highlight the effects of zeolite on the pathogenic bacteria and for modulating pathogenic bacterial populations.

## Funding

No funding was received for this manuscript.

## Conflicts of Interest

The authors declare no conflicts of interest.

## Data Availability

The data that support the findings of this study are available on request from the corresponding author. The data are not publicly available due to privacy or ethical restrictions.

## References

[1] Johar A. , Al-Thani N. , Al-Hadidi S. H. , Dlissi E. , Mahmoud M. H. , and Eltai N. O. , Antibiotic Resistance and Virulence Gene Patterns Associated with Avian Pathogenic *Escherichia coli* (APEC) from Broiler Chickens in Qatar, Antibiotics (Basel). (2021) 10, no. 5, 10.3390/antibiotics10050564. PMC8151107 34064966

[2] Hu J. , Afayibo D. J. A. , Zhang B. et al., Characteristics, Pathogenic Mechanism, Zoonotic Potential, Drug Resistance, and Prevention of Avian Pathogenic *Escherichia coli* (APEC), Frontiers in Microbiology. (2022) 13, 10.3389/fmicb.2022.1049391. PMC9793750 36583051

[3] Al-Anber M. and Al-Anber Z. , Utilization of Natural Zeolite as Ion-Exchange and Sorbent Material in the Removal of Iron, Desalination. (2008) 225, no. 1-3, 70–81, 10.1016/j.desal.2007.07.006.

[4] Rhodes C. J. , Properties and Applications of Zeolites, Science in Progress. (2010) 93, no. Pt 3, 223–284, 10.3184/003685010X12800828155007. PMC10365492 21047018

[5] Hedström A. , Ion Exchange of Ammonium in Zeolites: A Literature Review, Journal of Environmental Engineering. (2001) 127, no. 8, 673–681, 10.1061/(ASCE)0733-9372(2001)127:8(673).

[6] Sadeghi A. A. and Shawrang P. , Effects of Natural Zeolite Clinoptilolite on Passive Immunity and Diarrhea in Newborn Holstein Calves, Livestock Science. (2008) 113, no. 2-3, 307–310, 10.1016/j.livsci.2007.08.010.

[7] Toprak N. , Yilmaz A. , Ozturk E. , Yigit O. , and Cedden F. , Effect of Micronized Zeolite Addition to Lamb Concentrate Feeds on Growth Performance and Some Blood Chemistry and Metabolites, South African Journal of Animal Science. (2016) 46, no. 3, 313–320, 10.4314/sajas.v46i3.11.

[8] El-Nile A. , Elazab M. , Soltan Y. et al., Nano and Natural Zeolite Feed Supplements for Dairy Goats: Feed Intake, Ruminal Fermentation, Blood Metabolites, and Milk Yield and Fatty Acids Profile, Animal Feed Science and Technology. (2023) 295, no. 21, 10.1016/j.anifeedsci.2022.115522.

[9] Amad A. , The Effects of Natural Zeolites as Feed Additives on the Performance and Egg Quality in Old Laying Hen, Journal of poultry research. (2021) 18, no. 1, 13–18, 10.34233/jpr.919356.

[10] Abdelrahman M. M. , Al-Baadani H. H. , Qaid M. M. et al., Using Natural Zeolite as a Feed Additive in Broilers′ Diets for Enhancing Growth Performance, Carcass Characteristics, and Meat Quality Traits, Life. (2023) 13, no. 7, 10.3390/life13071548. PMC10382045 37511923

[11] Al-Faqieh M. A. , Abdelqader A. , and Aburjai T. , Effect of Different Levels of Aqueous Suspension of Bentonite Nanoparticles on Performance and Carcass Characteristics of Broiler Chickens, Jordan Journal of Agricultural Sciences. (2024) 20, no. 2, 141–148, 10.35516/jjas.v20i2.1055.

[12] Rahm C. , Detoxing & Remediating Land, Air, and Water & Implications on Human and Animal Health, PriMera Scientific Surgical Research and Practice. (2024) 4, no. 3, 77–81.

[13] Prasai T. P. , Walsh K. B. , Bhattarai S. P. et al., Zeolite Food Supplementation Reduces Abundance of Enterobacteria, Microbiology Research. (2017) 195, 24–30, 10.1016/j.micres.2016.11.006. 28024523

[14] Efsa , Villa R. E. , Azimonti G. et al., Safety and Efficacy of the Feed Additive Consisting of Clinoptilolite of Sedimentary Origin for all Animal Species for the Renewal of Its Authorisation (ZEOCEM, A.S.), EFSA Journal. (2025) 23, no. e9364, 10.2903/j.efsa.2025.9364. PMC11986687 40226503

[15] Pan D. and Yu Z. , Intestinal Microbiome of Poultry and Its Interaction with Host and Diet, Gut Microbes. (2014) 5, no. 1, 108–119, 10.4161/gmic.26945. 24256702 PMC4049927

[16] Johnson T. J. , Wannemuehler Y. , Doetkott C. , Johnson S. J. , Rosenberger S. C. , and Nolan L. K. , Identification of Minimal Predictors of Avian Pathogenic *Escherichia coli* Virulence for Use as a Rapid Diagnostic Tool, Journal of Clinical Microbiology. (2008) 46, no. 12, 3987–3996, 10.1128/JCM.00816-08. 18842938 PMC2593276

[17] Ewers C. , Janssen T. , Kiessling S. , Philipp H. C. , and Wieler L. H. , Rapid Detection of virulence-associated Genes in Avian Pathogenic *Escherichia coli* by Multiplex Polymerase Chain Reaction, Avian Diseases. (2005) 49, no. 2, 269–273, 10.1637/7293-102604R. 16094833

[18] Bhattarai R. K. , Basnet H. B. , Dhakal I. P. , and Alocilja E. C. , Virulence Genes of Avian Pathogenic *Escherichia coli* Isolated from Commercial Chicken in Nepal, Comparative Immunology, Microbiology and Infectious Diseases. (2023) 95, 10.1016/j.cimid.2023.101961. 36870115

[19] Gao Q. , Wang X. , Xu H. et al., Roles of Iron Acquisition Systems in Virulence of Extraintestinal Pathogenic *Escherichia coli*: Salmochelin and Aerobactin Contribute More to Virulence than Heme in a Chicken Infection Model, BMC Microbiology. (2012) 12, no. 1, 10.1186/1471-2180-12-143. PMC3496646 22817680

[20] Guragain M. , Bagi L. , Liu Y. , and Bosilevac J. M. , Characterization of Extraintestinal Pathogenic *Escherichia coli* from Human Clinical and Poultry Samples, Microorganisms. (2025) 13, no. 11, 10.3390/microorganisms13112603. PMC12654797 41304289

[21] Panaiotov S. , Tancheva L. , Kalfin R. , and Petkova-Kirova P. , Zeolite and Neurodegenerative Diseases, Molecules. (2024) 29, no. 11, 10.3390/molecules29112614. PMC11173861 38893490

[22] Kitamoto S. , Nagao-Kitamoto H. , Kuffa P. , and Kamada N. , Regulation of Virulence: the Rise and Fall of Gastrointestinal Pathogens, Journal of Gastroenterology. (2016) 51, no. 3, 195–205, 10.1007/s00535-015-1141-5. 26553054 PMC4767578

[23] Broom L. J. , Monteiro A. , and Pinon A. , Recent Advances in Understanding the Influence of Zinc, Copper, and Manganese on the Gastrointestinal Environment of Pigs and Poultry, Animals. (2021) 11, no. 5, 10.3390/ani11051276. PMC8145729 33946674

[24] Qiu K. , Chen J. , Zhang G. et al., Effects of Dietary Crude Protein and Protease Levels on Performance, Immunity Capacity, and AA Digestibility of Broilers, Agriculture. (2023) 13, no. 3, 10.3390/agriculture13030703.

[25] Al-musawy S. A. A.-a. , Mohammed M. F. , and Mohammed H. J. , Effect of Adding Zeolite to the Diet in Health Status of Broiler Infected With Pathogenic *E. coli* O157: H7, Acta BioMedica. (2023) 94, no. 3.

[26] Ostovar G. and Boedicker J. Q. , Phenotypic Memory in Quorum Sensing, PLoS Computational Biology. (2024) 20, no. 7, 10.1371/journal.pcbi.1011696. PMC11257393 38976753

[27] Hananeh W. , Alajlouni A. , Al- Rukibat R. , and Al-Alajlouni M. , Toxicological Evaluation of Locally Sourced Jordanian Zeolite as a Safe Feed Additive in Broiler Diets: Histopathological and Hematological Perspectives, Iraqi Journal of Veterinary Sciences, In Press.

[28] Dunislawska A. , Biesek J. , Banaszak M. , Siwek M. , and Adamski M. , Effect of Zeolite Supplementation on Gene Expression in the Intestinal Mucosa in the Context of Immunosafety Support in Poultry, Genes. (2022) 13, no. 5, 10.3390/genes13050732. PMC9140869 35627116

[29] Haneneh W. , Alajlouni M. A. , Al-Rukibat R. , and Al- Alajlouni G. M. , Toxicological Evaluation of Locally Sourced Jordanian Zeolite as a Safe Feed Additive in Broiler Diets: Histopathological and Hematological Perspectives, Iraqi Journal of Veterinary Sciences. (2026) 40, no. 1, 213–224, 10.33899/ijvs.2025.164588.4482.

